# Effect of Mechanical Stress on Cotton Growth and Development

**DOI:** 10.1371/journal.pone.0082256

**Published:** 2013-12-16

**Authors:** Zhiyong Zhang, Xin Zhang, Sufang Wang, Wanwan Xin, Juxiang Tang, Qinglian Wang

**Affiliations:** School of Life Science and Technology, Henan Institute of Science and Technology, Xinxiang, Henan, China; East Carolina University, United States of America

## Abstract

Agricultural crops experience diverse mechanical stimuli, which may affect their growth and development. This study was conducted to investigate the effects of mechanical stresses caused by hanging labels from the flower petioles (HLFP) on plant shape and cotton yields in four cotton varieties: CCRI 41, DP 99B, CCRC 21, and BAI 1. HLFP significantly reduced plant height by between 7.8% and 36.5% in all four lines and also significantly reduced the number of fruiting positions per plant in the CCRI 41, DP 99B and CCRC 21 lines. However, the number of fruiting positions in BAI 1 was unaffected. HLFP also significantly reduced the boll weight for all four cultivars and the seed cotton yields for CCRI 41, DP 99B and BAI 1. Conversely, it significantly increased the seed cotton yield for CCRC 21 by 11.2%. HLFP treatment did not significantly affect the boll count in the fruiting branches of the 1^st^ and 2^nd^ layers in any variety, but did significantly reduce those on the 3^rd^ and 4^th^ fruiting branch layers for CCRI 41 and DP 99B. Similar trends were observed for the number of bolls per FP. In general, HLFP reduced plant height and boll weight. However, the lines responded differently to HLFP treatment in terms of their total numbers of fruiting positions, boll numbers, seed cotton yields, etc. Our results also suggested that HFLP responses might be delayed for some agronomy traits of some cotton genotypes, and that hanging labels from early-opening flowers might influence the properties related with those that opened later on.

## Introduction

Plants are immobile and therefore unable to escape from threats or unfavorable environments. Consequently, they have evolved diverse mechanisms for coping with and mitigating the effects of various stresses [1]. The adverse effects of disadvantageous environmental conditions, such as excessively high or low temperatures, salt levels, and drought on plant growth and development have been extensively documented. However, the effects of mechanical stresses caused by factors such as wind, rain, physical contact, wounding, and gravity on plant growth and development have not been studied in such detail [2],[3].

Agricultural plants are subject to many different kinds of mechanical stress, including shaking [4],[5] and bending of their stems [6]–[9], rubbing of their stems with fingers [10],[11], brushing [12], spraying with water [13],[14], mechanical vibration [15], and stress caused by running water [16]. All of these stresses affect plant morphology and development [17].

Different plant species respond to mechanical stress in different ways. Some plants, such as *Mimosa pudica*, respond rapidly via specialized responsive mechanisms, but others respond slowly [2],[18]. In some cases, mechanical stress causes visible phenotypic changes. For example, it has been shown that touching can inhibit growth and retard flowering in *Arabidopsis* [18]. Similarly, touching was found to reduce the height of cotton plants but did not significantly affect their flowering, the number of bolls they produced, or the cotton yield [4].

In cotton production, it is common to hang labels on flower petioles in order to record the times at which they blossomed and started forming bolls. The mechanical stimulus caused by this practice may affect the growth and development of the cotton plant, but its potential impact has not previously been investigated. Therefore, the work reported herein was conducted to investigate the responses of different cotton genotypes to the mechanical stress caused by hanging labels from cotton flower petioles, in terms of plant shape and yield.

## Materials and Methods

### Cotton cultivars

Four commercialized transgenic insect-resistant cotton cultivars, CCRI41, DP 99B, CCRC21 and BAI1, were used in this study. CCRI41 was bred by the Cotton Research Institute of the Chinese Academy of Agricultural Sciences; CCRC21 was bred by the Cotton Research Center of the Shandong Academy of Agricultural Sciences; DP 99B was bred by Monsanto Company; and BAI1 was bred by Henan Institute of Sciences and Technology.

In China, transgenic insect-resistant cotton cultivars are widely adopted in the yield in recent years. Maybe, insect-resistant gene transformation could result in the different responses of cotton plants to mechanical stress, but it was not in the research scopes of this experiment. Therefore, only transgenic cotton cultivars were used in this experiment. All four cultivars have growth periods of around 130 days. However, CCRI41 and DP 99B cultivars often pre-maturely senesced, BAI1 was resistant to pre-mature senescence, and CCRC21 was in between them.

### Field experiment

The four cotton cultivars were planted in a sandy loam soil with a pH of 8.5 (water: soil  =  5 1), an organic matter content of 0.60% (determined by digestion with potassium dichromate under strongly acidic conditions), an available nitrogen content of 18.6 mg kg^−1^ (determined by extraction with 1 M KCl), an available P content of 16.2 mg kg^−1^ (determined by extraction with 0.5 M NaHCO_3_), and an available K content of 158.5 mg kg^−1^ (determined by extraction with 1 M NH_4_OAC). Planting was conducted in 2009 and 2010 at the experimental field station (35°16′N; 113°56′E) of the Henan Institute of Science and Technology, Xinxiang, Henan Province.

The cotton seeds were sowed under plastic film mulching, respectively, on April 26^th^, 2009 and 2010, according to a random block design with four biological replicates. Each block contained 4 plots, and each plot was planted with only one cultivar. Each plot contained four 10 m long rows with an inter-row spacing of 0.8 m and an intra-row spacing of 0.27 m. The planting density was 45,000 plants per hm^2^.

Conventional agricultural practices were applied in the study. 150 kg N, 100 kg P and 75 kg K in the form of urea, diammonium phosphate and potassium sulphate, respectively, were applied per hm^2^ before sowing. In the early flowering stage, additional quantities of urea-N (150 kg N per hm^2^) and K (75 kg K per hm^2^) were applied by top-dressing. All plots were treated with chemical pesticides to keep insects away.

### Hanging labels from flower petioles (HLFP)

During early flowering season, 5 adjacent plants in one of the central rows of each plot were selected and tagged with labels that were hung from their flower petioles during 9–10 A.M every day and kept in place throughout the blossoming stage. HLFP was made for each flower at its opening day. The anthesis and boll-opening dates for the tagged plants were recorded on their labels.

A label with a thin thread at its one end weighted 337±20 (mean±SD) mg with length of 3.5 cm, width of 3.0 cm and thickness of 2.2 mm. The label was tied to flower petiole by thin thread and vertically suspended when tagging. The labels are widely used in field experiment of cotton.

### Measurement of plant shape parameters and yield and fiber quality traits

During the harvest season, the numbers of fruiting branches (FB) and fruiting positions (FP) on each plant were counted; the height of the stem (from base to tip) was measured, along with the length of each fruiting branch and the location of each fruiting position. The fruiting branches were recorded as the 1^st^ FB, 2^nd^ FB, *etc*, from the bottom. The fruiting positions were numbered horizontally, as the 1^st^ FP, 2^nd^ FP, etc. from the stem. The fruiting position length was defined as the distance between the 1^st^ FP and the main stem or between adjacent FPs; the distance between the stem and the 1^st^ FP was recorded as the 1^st^ FP length (FPL), while that between the 1^st^ and 2^nd^ FPs was recorded as the 2^nd^ FPL, and so on. The fruiting branch length (FBL) was calculated as the sum of all FPLs on a single branch, *i.e*., the distance between the main stem and the terminal FP.

We defined four ‘layers’ of fruiting branches: the first layer consisted of the 1^st^ to 4^th^ FBs, the second layer consisted of the 5^th^ to 8^th^, the third layer consisted of the 9^th^ to 12^th^, and all FBs from the 13^th^ upwards were assigned to the fourth layer. All fruiting positions more distal than the 3^rd^ FP on a given fruiting branch were collectively referred to as >3^rd^ FPs. The number of bolls, fruiting branches and fruiting position lengths within each FB layer were calculated. Since many plants had fewer than 13 fruiting branches and many fruiting branches had fewer than three fruiting positions, data for the fourth fruiting branch layer and for fruiting position numbers greater than three on any given branch were not subjected to statistical analysis.

Cotton was harvested twice, on September 22^nd^ and October 22^nd^. All opened bolls were harvested and counted. The harvested cotton was weighed as seed cotton yield per plant. Boll weight was average seed cotton weight of all harvested opened bolls in one plant. Ginned cotton was used for determination of fiber quality traits such as fiber length, fiber strength, micronaire, uniformity index and fiber elongation with high volume instrument (HVI).

### Biostatistical analyses

The experiment was first conducted in 2009 and then repeated in 2010, with both replicates yielding similar results. In both cases, 20 control and treated plants of each genotype were studied, and were distributed evenly among the four experimental plots. The two-year experimental results were pooled and subjected to statistical analysis by means of analysis of variance (ANOVA), and Student's *t*-test was used to identify significant differences (at the *p*<0.05 level) between the mean results for different treatments and those observed for control plants.

## Results

### Effect of HLFP on cotton plant shape

As shown in Table 1, HLFP significantly reduced the stem height in all four genotypes relative to control plants by between 7.8% and 36.5%. HLFP also significantly reduced the FB number for the DP 99B variety, but had no significant effect on FB numbers in other genotypes. Furthermore, HLFP significantly reduced the total number of fruiting positions in the CCRI 41, DP 99B and CCRC 21 varieties by 33.3%, 37.4% and 18.8%, respectively, but had no effect on the fruiting position count in BAI 1 (Table 1).

**Table 1:** Plant heights, numbers of fruiting branches, and numbers of fruiting positions for four cotton genotypes that were subjected to mechanical stress caused by hanging labels from the petioles (HLFP) of every white flower in the field.

Genotypes	Treats	Plant height (cm)	Fruiting branches (No.plant^−1^)	Fruiting positions (No. plant^−1^)
CCRI41	Control	96.6±7.5 a	14.4±2.3 a	59.9±13.5 a
	HLFP	68.5±5.4 b	13.1±1.5 a	40.2±6.7 b
DP 99B	Control	86.4±5.2 a	15.1±1.0 a	78.0±6.2 a
	HLFP	54.9±4.8 b	12.4±2.1 b	48.8±11.4 b
BAI1	Control	94.7±4.3 a	13.1±0.6 a	52.6±5.5 a
	HLFP	79.3±4.1 b	13.1±1.6 a	47.9±3.5 a
CCRC21	Control	96.8±3.4 a	13.0±0.9 a	51.5±5.3 a
	HLFP	89.2±3.0 b	12.4±2.5 a	41.8±10.4 b

For each genotype, values (mean±SD) in the same column that are followed by different lower case letters differ significantly at the *p*<0.05 level. n = 40 plants in all cases.

HLFP significantly reduced the total FBL of all four varieties by between 14.0% and 38.1% (Table 2). More specifically, the length of the fruiting branches in the 1^st^, 2^nd^ and 3^rd^ layers was reduced by 22.1%, 29.5% and 47.9%, respectively, for CCRI 41; by 35.4%, 30.7% and 50.7%, respectively, for DP 99B; by 16.4%, 23.1% and 41.2%, respectively, for BAI 1; and by 4.5%,8.3%,35.4%, respectively, for CCRC 21 (Table 2).

**Table 2:** Effects of mechanical stress caused by hanging labels from the petioles (HLFP) of every white flower in the field on the fruiting branch lengths (FBL values) for four cotton genotypes.

Genotypes	Treats	1^st^ Layer	2^nd^ Layer	3^rd^ Layer	Mean
		FBL (cm)			
CCRI41	Control	33.5±3.9 a	37.6±5.8 a	28.8±6.8 a	33.3±5.3 a
	HLFP	26.1±3.1b	26.5±3.5 b	15.0±3.8 b	22.5±3.2 b
DP 99B	Control	35.3±3.1 a	38.1±3.6 a	28.4±2.8 a	33.9±2.3 a
	HLFP	22.8±2.5 b	26.4±2.8 b	14.0±3.2 b	21.0±1.6 b
BAI1	Control	35.3±3.2 a	38.1±2.8 a	28.4±3.5 a	33.9±2.4 a
	HLFP	29.5±2.5 b	29.3±1.9 b	16.7±1.7 b	25.1±1.7 b
CCRC21	Control	31.0±2.8 a	37.4±3.6 a	23.7±2.4 a	30.7±2.2 a
	HLFP	29.6±3.1 a	34.3±2.8 a	15.3±5.4 b	26.4±3.3 a

For each genotype, values (mean±SD) in the same column that are followed by different lower case letters differ significantly at the *p*<0.05 level. n = 40 plants in all cases.

HLFP also significantly reduced the FPL values in all four varieties by between 8.2% and 22.9% (Table 3). More specifically, HLFP reduced the 1^st^, 2^nd^ and 3^rd^ FPL values by 26.6%, 19.8% and 20.5%, respectively, for CCRI 41; by 18.1%, 17.9% and 28.6%, respectively, for DP 99 B; by 15.7%, 20.7% and 27.0%, respectively, for BAI 1; and by 7.9%, 8.9% and 6.3%, respectively, for CCRC 21 (Table 3). In CCRI 41, DP 99B and BAI 1, the most pronounced reductions in FPL occurred between the second and third fruiting positions. Conversely, in CCRC 21, all three FPL values were reduced by similar amounts, although the reduction was least pronounced for the third FP (Table 3).

**Table 3:** Effects of mechanical stress caused by hanging labels from the petioles (HLFP) of every white flower in the field on the fruiting position lengths (FPL values) for four cotton genotypes.

Genotypes	Treats	1^st^	2^nd^	3^rd^	Mean
		FPL (cm)			
CCRI41	Control	12.5±0.8 a	8.6±0.7 a	6.8±1.0 a	9.3±0.6 a
	HLFP	9.4±1.1 b	6.9±0.9 b	5.4±0.7 b	7.2±0.8 b
DP 99B	Control	9.4±1.3 a	7.8±0.8 a	7.0±0.9 a	7.8±0.7 a
	HLFP	7.7±1.0 b	6.4±1.4 b	5.0±0.9 b	6.2±0.8 b
BAI1	Control	11.4±0.4 a	8.2±0.5 a	7.4±1.0 a	8.9±0.5 a
	HLFP	9.6±0.6 b	6.5±0.5 b	5.4±0.3 b	7.2±0.4 b
CCRC21	Control	11.3±0.6 a	7.9±0.6 a	6.3±0.7 a	8.5±0.4 a
	HLFP	10.4±0.7 a	7.2±0.4 a	5.9±0.8 a	7.8±0.4 a

For each genotype, values (mean±SD) in the same column that are followed by different lower case letters differ significantly at the *p*<0.05 level. n = 40 plants in all cases.

### Effects of HLFP on cotton plant yield and fiber quality

In the CCRI 41 and DP 99B varieties, HLFP significantly reduced the number of bolls per plant by 34.8% and 27.6%, respectively. However, it did not affect boll numbers in BAI 1, and significantly increased them for CCRC 21. HLFP significantly reduced boll weights by between 10.6% and 14.3% in all four varieties, and also significantly reduced the seed cotton yields for the CCRI 41, DP 99B and BAI 1 varieties by 44.3%, 38.5% and 9.3%, respectively. However, it significantly increased the cotton seed yield by 11.2% for CCRC 21 (Table 4).

**Table 4:** Effects of mechanical stress caused by hanging labels from the petioles (HLFP) of every white flower in the field on the number of bolls, boll weight and seed cotton yield for four cotton genotypes.

Genotypes	Treats	Bolls (No.Plant^−1^)	Boll weight (g)	Seed cotton yield (g plant^−1^)
CCRI41	Control	20.4±1.1 a	4.9±0.2 a	99.5±12.9 a
	HLFP	13.3±0.6 b	4.2±0.1 b	55.4±6.3 b
DP 99B	Control	23.9±2.21 a	4.9±0.3 a	117.0±10.8 a
	HLFP	17.3±2.20 b	4.2±0.2 b	72.0±10.3 b
BAI1	Control	16.6±1.1 a	4.4±0.2 a	73.8±3.3 a
	HLFP	17.0±1.0 a	3.9±0.1 b	66.9±4.2 b
CCRC21	Control	13.0±1.1 b	4.7±0.3 a	61.6±3.3 b
	HLFP	16.3±1.2 a	4.2±0.2 b	68.5±2.3 a

For each genotype, values (mean±SD) in the same column that are followed by different lower case letters differ significantly at the *p*<0.05 level. n = 40 plants in all cases.

HLFP had no significant effects on the number of bolls on the fruiting branches of the 1^st^ and 2^nd^ layers for CCRC 41, DP 99B and BAI 1, but significantly increased the number of bolls in these layers by 68.3% and 24.5%, respectively, in CCRC 21. HLFP significantly reduced the number of bolls on the fruiting branches of the 3^rd^ and 4^th^ layers in CCRI 41 and DP 99B but did not significantly affect the boll count in the 3^rd^ and 4^th^ layers in BAI 1 and CCRC 21 (Table 5).

**Table 5:** Effects of mechanical stress caused by hanging labels from the petioles (HLFP) of every white flower in the field on the number of bolls found on fruiting branch (FB) layers in four cotton genotypes.

Genotype	Treat	1^st^ Layer FB	2^nd^ Layer FB	3^rd^ Layer FB	>3^rd^ Layer FB
		Bolls (No.)			
CCRI41	Control	7.6±0.8 a	6.3±1.5 a	4.2±1.1 a	2.2±0.8 a
	HLFP	6.4±1.0 a	4.2±0.8 a	1.8±0.7 b	0.5±0.4 b
DP 99B	Control	7.2±1.0 a	7.8±1.1 a	6.1±1.1 a	2.8±1.0 a
	HLFP	7.5±1.4 a	6.1±1.5 a	2.4±1.3 b	0.8±0.6 b
BAI1	Control	5.5±0.8 a	5.9±1.4 a	3.9±1.0 a	1.4±0.8 a
	HLFP	6.8±1.2 a	5.4±1.0 a	3.3±0.8 a	1.3±0.6 a
CCRC21	Control	4.1±0.7 b	4.9±1.0 a	3.4±1.2 a	0.6±0.4 a
	HLFP	6.9±1.4 a	6.1±1.4 a	2.8±0.9 a	0.4±0.3 a

For each genotype, values (mean±SD) in the same column that are followed by different lower case letters differ significantly at the *p*<0.05 level. n = 40 plants in all cases.

HLFP had no significant effect on the boll counts for the 1^st^ and 2^nd^ fruiting positions in any of the four studied genotypes, or on those of 3^rd^ FP and >3^rd^ FPs for BAI 1 and CCRC 21. However, it significantly reduced the boll counts for the 3^rd^ and >3^rd^ FPs in CCRI 41 and DP 99B by between 31.7% and 93.8% (Table 6).

**Table 6:** Effects of mechanical stress caused by hanging labels from the petioles (HLFP) of every white flower in the field on the number of bolls retention on different fruiting positions (FP) in four cotton genotypes.

Genotype	Treat	1^st^ FP	2^nd^ FP	3^rd^ FP	>3^rd^ FP
		Bolls (No.)			
CCRI41	Control	8.9±1.3 a	5.5±0.8 a	2.6±0.4 a	1.6±0.5 a
	HLFP	7.6±1.0 a	3.8±0.9 a	1.3±0.3 b	0.1±0.2 b
DP 99B	Control	9.6±1.4 a	6.6±1.4 a	4.1±0.7 a	3.5±0.8 a
	HLFP	7.5±1.1 a	5.3±0.6 a	2.8±0.8 b	0.9±0.7 b
BAI1	Control	8.6±0.9 a	4.9±0.9 a	2.9±0.8 a	0.4±0.3 a
	HLFP	9.2±0.8 a	5.1±1.3 a	2.7±0.7 a	0.4±0.4 a
CCRC21	Control	6.1±0.8 a	4.3±0.5 a	1.6±0.7 a	1.0±0.7 a
	HLFP	7.5±0.1 a	5.1±0.8 a	2.8±0.5 a	0.9±0.4 a

For each genotype, values (mean±SD) in the same column that are followed by different lower case letters differ significantly at the *p*<0.05 level. n = 40 plants in all cases.

HLFP had no significant effects on fiber quality indexes such as firber length, uniformity index, fiber elongation and fiber strength in four cotton genotypes. HLFP decreased micronaire in increased magnitude of CCRI41>DP99B>CCRC21>BAI1, however, significantly only for CCRC41 (Table 7).

**Table 7:** Effects of mechanical stress caused by hanging labels from the petioles (HLFP) of every white flower in the field on fiber quality expressed as fiber length, uniformity index, micronaire, fiber elongation and strength in four cotton genotypes.

Genotype	Treat	Fiber length (mm)	Uniformity index (%)	Micronaire	Fiber elongation (%)	Fiber strength
CCRI41	Control	28.9±0.9 a	83.3±1.3 a	5.1±0.3 a	6.6±0.2 a	27.1±1.2 a
	HLFP	28.0±0.9 a	82.7±1.8 a	3.9±0.6 b	6.8±0.2 a	26.5±1.8 a
DP 99B	Control	28.7±0.8 a	82.9±1.8 a	5.2±0.4 a	6.5±0.2 a	27.1±1.2 a
	HLFP	28.1±1.0 a	82.9±1.6 a	4.4±0.7 a	6.7±0.3 a	26.7±1.8 a
BAI1	Control	27.8±0.9 a	82.5±1.5 a	4.7±0.5 a	6.7±0.2 a	25.4±0.9 a
	HLFP	27.7±1.0 a	83.6±1.3 a	4.6±0.6 a	6.6±0.3 a	26.6±1.1 a
CCRC21	Control	29.5±1.2 a	84.1±1.5 a	5.0±0.3 a	6.6±0.2 a	26.7±1.2 a
	HLFP	28.1±1.1 a	83.4±2.0 a	4.3±0.6 a	6.7±0.3 a	26.2±1.9 a

For each genotype, values (means±D) in the same column that are followed by different lower case letters differ significantly at the *p*<0.05 level. n = 40 plants in all cases.

## Discussion

### HLFP caused mechanical stress and induced thigmomorphogenesis

Plants respond to environmental stresses in different ways, on both the physiological and developmental levels, and undergo diverse changes to adapt to their surroundings. For example, in windy environments, they became short and strong [19],[20]. Similarly, soybeans and sunflowers produced larger quantities of xylem and thicker cells when subjected to mechanical disturbance during growth [21]. In addition, it has been shown that trees grew taller when secured with ropes to reduce mechanical stresses caused by wind [22]. The processes that give rise to such adaptations are collectively referred to as thigmomorphogenesis [23],[24].

Plants are both responsive and highly sensitive to mechanical stimuli. Tendrils responded to stimuli caused by weights of 1–5 mg [25], and even to gentler stimuli with only 0.25 mg [26]. Our results showed that hanging labels around flower petioles formed mechanical stress and significantly reduced stem height in four cotton varieties (Table1). Similar results have previously been reported in cotton by handling plants [4], *Arabidopsis* by bending leaves back and forth by hand [18] and tomato by brushing shoots [27], though, with different mechanical stresses.

#### The different responses of the four cotton genotypes to HLFP

The four cotton varieties examined in this work differed in the magnitude of their responses to mechanical stimulus via HLFP. In tomatoes, similar mechanical stresses reduced stem heights by 43% to 29% for the “Sunny” variety but only by 37% to 17% for the “Wolfpack” variety [27]. Our results indicated that the cotton cultivars (CCRI41 and DP 99B) susceptible to pre-mature senescence were more sensitive to HLFP than the other two that were investigated.

Different mechanical stimuli have different effects on vegetative and reproductive growth. In one study, electronic shaking reduced the heights of peach trees by 80% [28]; while this did not affect fruit weight, it did reduce flowering density and fruit growth by changing the angle between the fruiting branches and the main stem [29]. Some plants respond positively to mechanical stimuli [17]. For example, mechanical stimulation enhanced branch growth in both *Arabidopsis* [30] and *Potentilla reptans* L. [31]. Similarly, “brushing” promoted leaf thickening and general growth in cauliflowers, lettuce and celery [12].

Different responses from different mechanical stresses were further verified. Frizzell and colleagues (1960) reported that manual shaking of plants had no significant effects on cotton boll growth [4]. However, in this study, we found that HLFP not only significantly decreased boll weight of four cotton genotypes, but also induced different effects in different cultivars, with respect to both boll number and seed cotton yield. This might result from ethylene release amount or sensitivity. Mechanical stresses induced ethylene release and changed growth and development [32]. However, the ability to produce ethylene in response to mechanical stresses and the types of responses differed between different genotypes of the same species [33],[34].

#### Cumulative effects of HLFP

In some cases, plants may exhibit delayed responses to stress [35]. In cotton, flowers on lower fruiting branches open before those at higher levels (i.e. those on the 1^st^ FB open first, followed by the 2^nd^ FB, etc.), and flowers closer to the stem open before those at more distant positions (i.e. those at the 1^st^ FP open before those at the 2^nd^, etc.). The labels used in this work were therefore attached in the same temporal order by daily labeling detailed in “materials and methods”, which may have caused memory effects whereby FBL of higher fruiting branch layers and boll number on higher fruiting branches or at more distal flowering points would have been influenced by the mechanical stress experienced by previously flowers-tagged labels. This could potentially have increased the magnitude of their HLFP responses.

HLFP had increasingly severe effects on the 1^st^, 2^nd^, and 3^rd^ fruiting branch layers length in all four studied genotypes (Table 2). However, in terms of boll number, HLFP exhibited cumulative effects only for CCRI 41 and DP 99B. HLFP had no significant effects on the boll numbers for the 1^st^ and 2^nd^ FB layers in the CCRI 41 and DP 99B cultivars but significantly reduced the boll numbers in the 3^rd^ and 4^th^ layers (Table 5). Similar delayed effects on boll numbers were observed as the FP number increased (Table 6). The cumulative effects of HLFP on boll number found expression in only CCRI41 and DP 99B, which might be related to senescent properties. CCRI41 and DP 99B senesced prematurely, compared with BAI1 and CCRI21.

### Conclusion

HLFP decreased plant height, the number of fruiting positions and boll weight in four cotton genotypes. However, different genotypes seemed to respond differently to HLFP with respect to variables such as plant height and seed cotton weight. In addition, some cotton plants may exhibit delayed responses in some agronomy traits to HLFP treatment.
